# *Wolbachia* Infections and Mitochondrial Diversity of Two Chestnut Feeding *Cydia* Species

**DOI:** 10.1371/journal.pone.0112795

**Published:** 2014-11-18

**Authors:** Dimitrios N. Avtzis, Vangelis Doudoumis, Kostas Bourtzis

**Affiliations:** 1 Laboratory of Forest Entomology, Forest Research Institute, Hellenic Agricultural Organization “Demeter”, Vassilika Thessaloniki, Greece; 2 Department of Environmental and Natural Resources Management, University of Patras, Agrinio, Greece; 3 Insect Pest Control Laboratory, Joint FAO/IAEA Division of Nuclear Techniques in Food and Agriculture, Vienna, Austria; University of Poitiers, France

## Abstract

*Cydia splendana* and *C. fagiglandana* are two closely related chestnut feeding lepidopteran moth species. In this study, we surveyed the bacterial endosymbiont *Wolbachia* in these two species. Infection rates were 31% in *C. splendana* and 77% in *C. fagiglandana*. MLST analysis showed that these two species are infected with two quite diverse *Wolbachia* strains. *C. splendana* with Sequence Type (ST) 409 from the A-supergroup and *C. fagiglandana* with ST 150 from the B-supergroup. One individual of *C. splendana* was infected with ST 150, indicating horizontal transfer between these sister species. The mitochondrial DNA of the two *Cydia* species showed a significantly different mtDNA diversity, which was inversely proportional to their infection rates.

## Introduction

*Wolbachia pipientis* are gram-negative Alphaproteobacteria. They are members of the family Rickettsiales, which are considered the most ubiquitous obligate intracellular symbionts reported so far in Arthropods [1]. Current estimates suggest that about 40% of all arthropod species may be infected with *Wolbachia* [2]. By establishing both somatic and gonadal infections, *Wolbachia* is able to manipulate many aspects of the biology, physiology, ecology and evolution of its hosts, including their reproductive properties [3]–[4]. In insects, *Wolbachia* has been reported to induce thelytokous parthenogenesis, feminization of genetic males, male killing, while the most abundant phenotype is cytoplasmic incompatibility (CI); however, *Wolbachia* infections with no obvious reproductive effect have also been detected [3]–[4]. All the above reproductive alterations favor an increase in infected females in host populations and thus the spread of *Wolbachia*, since the predominant mode of transmission of this symbiont is maternal [5].

Several studies suggest that *Wolbachia* infections can be transferred horizontally between different hosts. This is supported by the lack of congruence between host and symbiont phylogenetic trees [6]–[7]. Experimental evidence has been provided implicating parasitism, cannibalism and predation as potential routes for horizontal *Wolbachia* transfers in different systems [8]–[10]. Hybridization and introgression may have played a pivotal role in the movement of *Wolbachia* between closely related species [11]–[14]. In addition, ecological niche sharing could also be a driving force of horizontal transmission events [15].

During the last few years, many cases of mito-nuclear discordance have been reported in insect systems and, in most of them, *Wolbachia* has been identified as the driving factor. Being both maternally transmitted, mitochondrial DNA (mtDNA) and *Wolbachia* are in linkage disequilibrium. The spreading of a given *Wolbachia* strain will also result to the spreading of the associated mtDNA haplotypes (selective sweep), thus changing the frequency of the mtDNA haplotypes in a host population. Such selective sweeps have a significant impact on mtDNA, but not on nuclear DNA evolution and have to be considered in population, phylogenetic and phylogeographic studies [16]–[23].

The presence of *Wolbachia* in insect pests has implications for the management and control of these insects [24]–[25]. Population control of agricultural pests could be achieved with the Incompatible Insect Technique (IIT), which is based on the mechanism of *Wolbachia*-induced CI. The application of IIT requires, however, a perfect sexing system for male-only releases. In the absence of such a system, a combination of IIT with the Sterile Insect Technique (SIT) is recommended [26].

The chestnut feeding *Cydia* moths, *C. splendana* and *C. fagiglandana* comprise two of the most abundant and dangerous insect pests of sweet chestnut in European countries [27]. As a result, most pertinent studies so far aimed mainly at mapping their spatial distribution and refining control measures to reduce the damage caused by these pests [28]–[30]. However, trapping the two *Cydia* species using pheromones has been proven to be rather difficult [31]. Even the disentanglement of their distributions has been less than satisfactory, as these sympatrically occurring species not only resemble each other morphologically but also share similar life cycles [32]. DNA barcoding seems to provide the most precise approach to define their geographical distributions with accuracy [33].

Given that *Wolbachia* affects many aspects of the biology of its hosts and that it is a potential tool for pest control, we investigate here the prevalence of this symbiont in Greek populations of the two *Cydia* moth species. In addition, we genotype the detected bacterial strains via MLST and *wsp* gene-based approaches. We finally discuss the influence of *Wolbachia* infections on mitochondrial evolution and host population structure.

## Materials and Methods

### Sample collection, mtDNA barcoding and *Wolbachia* genotyping

**Figure 1:**
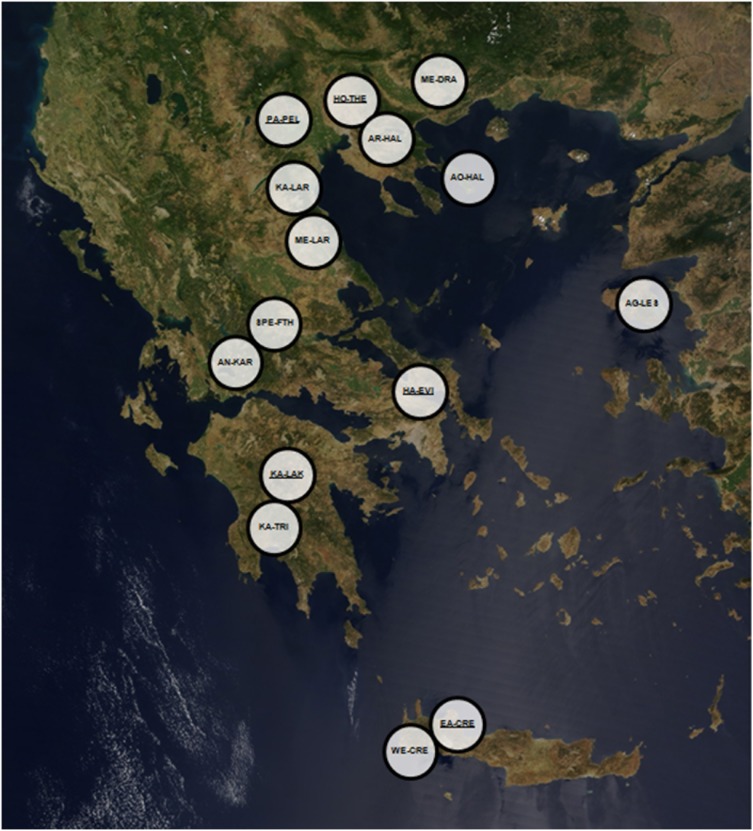
Distribution map of the sampled Greek populations (taken from NASA Earth Observatory–public domain). Underlined acronyms indicate those populations that participated in the MLST analysis.

Chestnuts suspected of moth infestation were collected from 15 chestnut-producing regions of Greece (Figure 1 and Table S1) and sent to the Forest Research Institute (Vassilika, Thessaloniki, Greece). For the collection of populations no specific permission was required while it did not involve any endangered, protected or threatened species. Chestnuts were manually opened and live larvae were placed individually into vials with 100% ethanol. From each of the 243 larvae, DNA was extracted using the GenElute Kit (Sigma) and processed following the manufacturer’s protocol. Amplification of an approximately 800 bp long locus from the 3′ end of mitochondrial *cytochrome oxidase I* (COI) gene was carried out with primers “Jerry” and “Pat” [34] in reactions containing 0.6 µl of MyTaq (BioLine, GmBH, Germany), 5 µl of the 5× MyTaq Red Reaction Buffer (BioLine, GmBH, Germany), 20 µ of each primer, 8 µl of DNA extract and ddH_2_O to a final volume of 25 µl. PCR conditions were: an initial denaturation step at 94°C for 3 minutes, followed by 40 cycles of 94°C for 30 s, 45°C for 30 s and 72°C for 1 minute, followed by a final extension step of 5 minutes at 72°C. PCR products were purified with the PureLink PCR Purification Kit (Invitrogen) and sequenced with an ABI 3730XL at CEMIA SA (Larissa, Greece) using both PCR primers.

A specific *16S rRNA* gene-based PCR assay was used for detection of *Wolbachia* with primers WspecF and WspecR resulting in an amplicon of 438 bp [35]. PCR amplifications were performed in 20 µl reaction mixtures containing 4 µl 5× reaction buffer (Promega), 25 mM MgCl2, deoxynucleotide triphosphate mixture (25 mM each), 25 µM of each primer, 0.1 U of Taq polymerase (Promega), 12.2 µl water and 1 µl of template DNA. The PCR protocol was: 35 cycles of 30 sec at 95°C, 30 sec at 54°C and 1 min at 72°C. The *Wolbachia* strains were genotyped with MLST- and *wsp* gene-based approaches. The *wsp* and MLST genes (*gatB*, *coxA*, *hcpA*, *ftsZ* and *fbpA*) were amplified using the respective primers reported in Baldo et al. [36] (Table S2). PCR amplifications were performed in 20 µl reaction mixtures containing 1× reaction buffer (Promega), 25 mM MgCl2, deoxynucleotide triphosphate mixture (25 mM each), 25 µM of each primer, 0.1 U of Taq polymerase (Promega), 12.2 µl water and 1 µl of template. PCR reactions were performed as follows: 5 min of denaturation at 95°C, followed by 35 cycles of 30 sec at 95°C, 30 sec at the appropriate temperature for each primer pair (52°C for *ftsZ*, 54°C for *gatB*, 55°C for *coxA*, 56°C for *hcpA*, 58°C for *fbpA* and *wsp*) and 1 min at 72°C. All reactions were concluded by a final extension step of 10 min at 72°C. For all PCR reactions described the appropriate negative (no DNA) and positive controls were included. All PCR were performed in triplicates. The PCR products were purified using a PEG (polyethylene glycol) - NaCl method [37]. Both strands of the products were sequenced using the corresponding forward and reverse primers. A dye terminator-labelled cycle sequencing reaction was carried out with the BigDye Terminator v3.1 Cycle Sequencing Kit (PE Applied Biosystems). Reaction products were analyzed using an ABI PRISM 310 Genetic Analyzer (PE Applied Biosystems).

### Phylogenetic analysis

*Wolbachia* sequences were manually edited with SeqManII by DNAStar and aligned using MUSCLE [38] and ClustalW [39], as implemented in Geneious 6.1.6 [40]. A final adjustment was done by eye. Phylogenetic analyses were performed using Maximum-Likelihood (ML) estimation and Bayesian Inference (BI) for a concatenated data set of the protein coding genes (*gatB*, *coxA*, *hcpA*, *ftsZ* and *fbpA*), as well as for the *wsp* gene. For the Maximum-Likelihood phylogeny, PAUP version 4.0b10 [41], as implemented in Geneious 6.1.6 [40], was used to select the optimal evolution model by critically evaluating the selected parameters, using the Akaike Information Criterion (AIC) [42]. For the concatenated data set and the *wsp* sequences, the submodels GTR+I+G and TVM+I+G were selected, respectively. The ML tree was constructed with 1000 bootstrap replications. Bayesian analyses were performed as implemented in MrBayes 3.1 [43]. The selected options were: random starting trees, four separate runs, each composed of four chains which were run for 6,000,000 generations; the first 20,000 generations were discarded and the cold chain was sampled every 100 generations. Posterior probabilities were computed for the remaining trees. All phylogenetic analyses were done with Geneious, version 6.1.6 [40].

MtDNA sequences were visualized using Chromas v. 1.45 and then aligned using Clustal X [44] with the default settings. Verified haplotypes were deposited in NCBI GenBank and referenced by haplotype designations provided below. Patterns of molecular diversity based on the mtDNA sequences were assessed by estimating haplotype (Hd), nucleotide diversity (π) [45] and the average number of nucleotide differences (k) [46] for every population using MEGA v.5 [47]. MEGA v.5 was also used in constructing the phylogenetic tree containing the *C. splendana* and *C. fagiglandana* haplotypes. For that, we employed the Neighbor Joining algorithm on the pair-wise Tamura-Nei [48] distances while the statistical support was assessed by 500 bootstrap replicates. Additionally, all populations were tested for the neutral mutation hypothesis with Tajima’s *D* and Fu’s *F* statistics [46], [49]–[50] using DNAsp version 5 [51]. All these parameters were also calculated for each species separately, complemented with the mismatch distribution plot estimated with DNAsp version 5 [51]. Finally, the correlation between genetic diversity (haplotype and nucleotide diversity) and *Wolbachia* prevalence was estimated with GenStat 12th Edition (supplied by VSN International).

### Nucleotide sequence accession numbers

All MLST, *wsp* and COI gene sequences generated in this study have been deposited into NCBI GenBank under accession numbers KJ139995–KJ140075 and KJ398246–KJ398313 respectively. The MLST and *wsp* gene sequences have been also deposited in the *Wolbachia* MLST database.

## Results

### *Wolbachia* in chestnut-feeding *Cydia* populations

A total of 147 field-collected adult insects from 14 populations of *C. splendana* and 96 field-collected adult insects from 11 populations of *C. fagiglandana* were tested. A significant difference was observed in the prevalence of *Wolbachia* between the two species (Table 1). *Wolbachia* infections were more prevalent in *C. fagiglandana* (77%) than in *C. splendana* (31%). This difference was not associated with the origin of the insects: cultivation or forest populations (data not shown). All populations of *C. fagiglandana* were infected while *Wolbachia* infection appeared to be absent from 2 out of 11 populations of *C. splendana* (Table 1). The prevalence of infection varied in the populations of both species ranging from 12.5 to 100% (Table 1).

**Table 1:** *Wolbachia* infection status in Greek *C. splendana* and *C. fagiglandana* populations. (n.d.: not detected).

		*Wolbachia* infection	
	Population	*Cydia splendana*	*Cydia fagiglandana*
**1**	**AO-HAL**	4/4 (100%)	12/16 (75%)
**2**	**KA-LAR**	2/3 (67%)	9/10 (90%)
**3**	**ME-DRA**	2/7 (28,6%)	2/7 (22,2%)
**4**	**EA-CRE**	n.d.	4/5 (80%)
**5**	**HO-THE**	1/1 (100%)	13/15 (86,67)
**6**	**ME-LAR**	3/20 (15%)	16/19 (84,2)
**7**	**KA-TRI**	5/40 (12,5%)	n.d.
**8**	**AG-LES**	4/16 (25%)	n.d.
**9**	**SPE-FTH**	3/7 (42,9%)	4/4 (100%)
**10**	**HA-EVI**	2/7 (28,6%)	n.d.
**11**	**WE-CRE**	1/2 (50%)	3/6 (50%)
**12**	**AN-KAR**	0/5 (0%)	3/4 (75%)
**13**	**KA-LAK**	16/28 (57,1%)	n.d.
**14**	**AR-HAL**	0/4 (0%)	2/2 (100%)
**15**	**PA-PEL**	3/3 (100%)	6/6 (100%)
**Total**		46/147 (31%)	74/96 (77%)

The *Wolbachia* strains present in nine adult insects originating from different *C. fagiglandana* populations (one forest- and two plantation-derived) and *C. splendana* populations (one forest- and two plantation-derived) were genotyped using MLST analysis. These samples were collected from different regions of Greece, as illustrated in Figure 1. As shown in Table 2, all *C. fagiglandana* specimens were found to be infected with the same B-supergroup *Wolbachia* strain with MLST gene alleles and Sequence Type (ST150) belonging to the clonal complex Sequence Type Complex 41 (STC-41). In addition, all these four specimens carried *Wolbachia* strains with an identical *wsp* protein profile (allele 10; Table 2). Four out of the five *C. splendana* specimens studied were infected with the same A-supergroup *Wolbachia* exhibiting new alleles for the MLST genes *coxA* (206), *hcpA* (241) and *fbpA* (396) and consequently a new sequence type (ST 409). In addition, a new *wsp* gene allele (674) was identified, closely related to allele 597, with new Hyper Variable Region (HVR) profile and HVR3 (262) and HVR4 (299) haplotypes (Table 2). The fifth *C. splendana* specimen (sample code 8E.1BK, originating from Paiko Pella or PA-PEL) carried the same B-supergroup *Wolbachia* strain detected in the *C. fagiglandana* samples (Table 2).

**Table 2:** MLST and wsp allele profiles of *Wolbachia* strains in Greek *Cydia* populations.

Samplecode	HostHaplotype	Supergroup	ST	MLST genes					*wsp*	HVR1	HVR2	HVR3	HVR4
				*gatB*	*coxA*	*hcpA*	*ftsZ*	*fbpA*					
7H.1AK	Fagi 8	B	150	16	14	40	36	4	10	10	8	10	8
6A.1AK	Fagi 8	B	150	16	14	40	36	4	10	10	8	10	8
5E.1AK	Fagi 6	B	150	16	14	40	36	4	10	10	8	10	8
2C.1AK	Fagi 5	B	150	16	14	40	36	4	10	10	8	10	8
8E.1BK	Splenda12	B	150	16	14	40	36	4	10	10	8	10	8
5G.1BK	Splenda12	A	409	175	206	241	140	396	674	212	28	262	299
4E.1BK	Splenda47	A	409	175	206	241	140	396	674	212	28	262	299
3G.1BK	Splenda12	A	409	175	206	241	140	396	674	212	28	262	299
9F.2AK	Splenda12	A	409	175	206	241	140	396	674	212	28	262	299

Identical nucleotide sequences at a given locus for different strain were assigned the same arbitrary allele number. Each strain was then identified by the combination of the five MLST allelic numbers, representing its allelic profile. Each unique allelic profile was assigned an ST (Sequence Type), which ultimately characterizes a strain [53]. Wsp profiles are shown in the last left column, respectively. (HVR: Hyper Variable Region).

**Figure 2:**
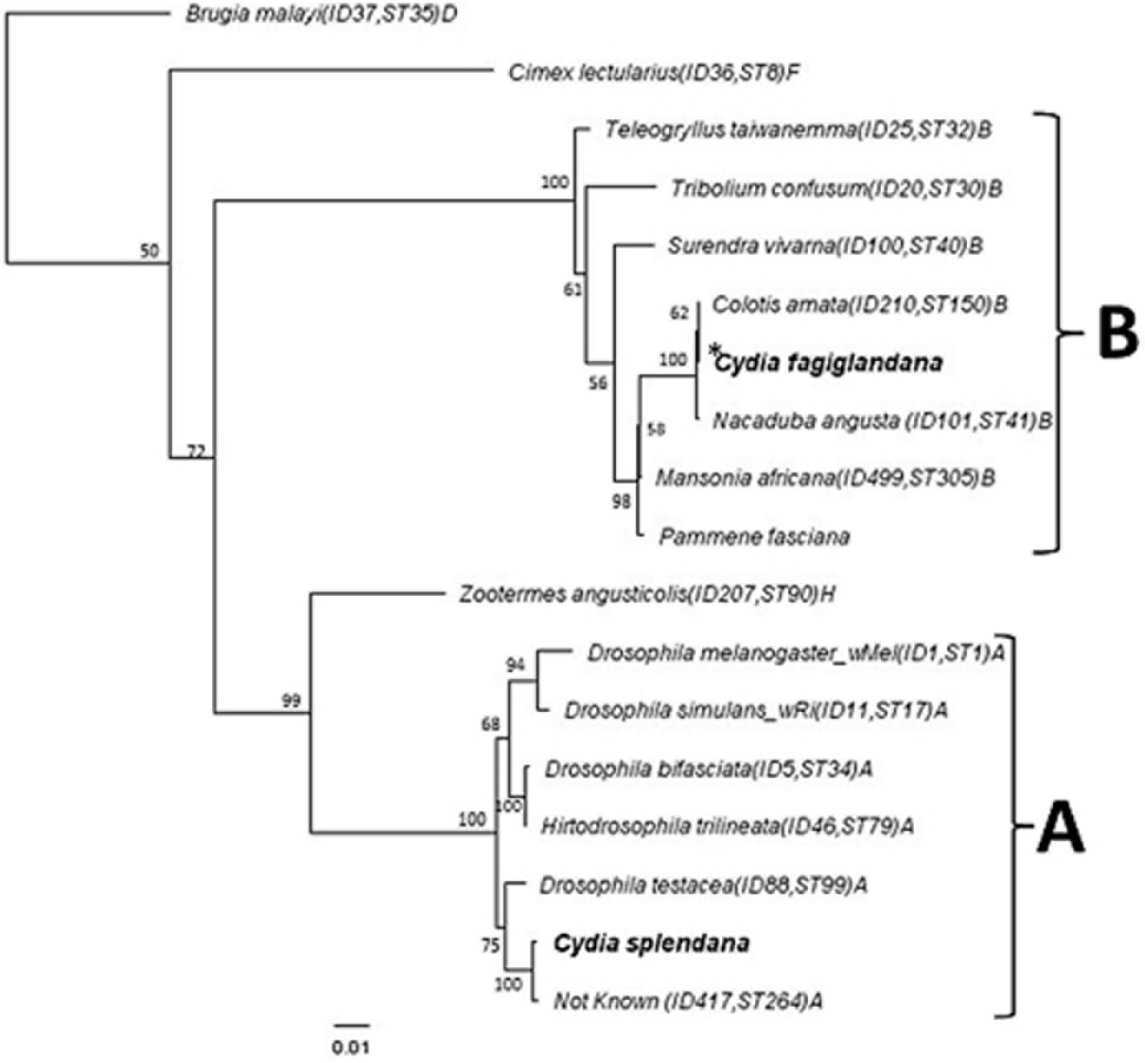
Maximum Likelihood inference phylogeny based on the concatenated MLST data (2,079 bp or 2073 bp). The two *Wolbachia* strains present in *Cydia* are indicated in bold letters; the other strains represent supergroups A, B, D, F and H. Strains are characterized by the names of their host species, the ID code and the ST number from the MLST database (excluding the strain of *Pammene fasciana*, unpublished data). *Wolbachia* supergroups are shown to the right of the host species names. ML bootstrap values based on 1000 replicates are given (only values>50% are indicated). *This *Wolbachia* stain was detected in all C. *fagiglandana* and in the *C. splendana* (8E. 1BK) specimens.

Phylogenetic analysis based on the concatenated dataset of all MLST loci revealed that the *Wolbachia* strain infecting all *C. fagiglandana* specimens and one of *C*. *splendana* (8E.1BK, PA-PEL population) belong to supergroup B, while the *Wolbachia* strain infecting the other four *C. splendana* specimens is a member of supergroup A, as shown in the Maximum Likelihood tree presented in Figure 2. The MLST analysis also showed that the *Cydia Wolbachia* strain of supergroup B is member of the common clonal complex STC-41 while the A supergroup *Wolbachia* strain infecting *C. splendana* (excluding the population PA-PEL) clusters with ST 99 and ST 264 strains (Figure 2). Phylogenetic analysis based on the *wsp* gene confirmed the MLST-based data (data not shown). MLST- and *wsp* gene-based Bayesian phylogenetic analysis provided identical results.

### Mitochondrial DNA analysis

**Figure 3:**
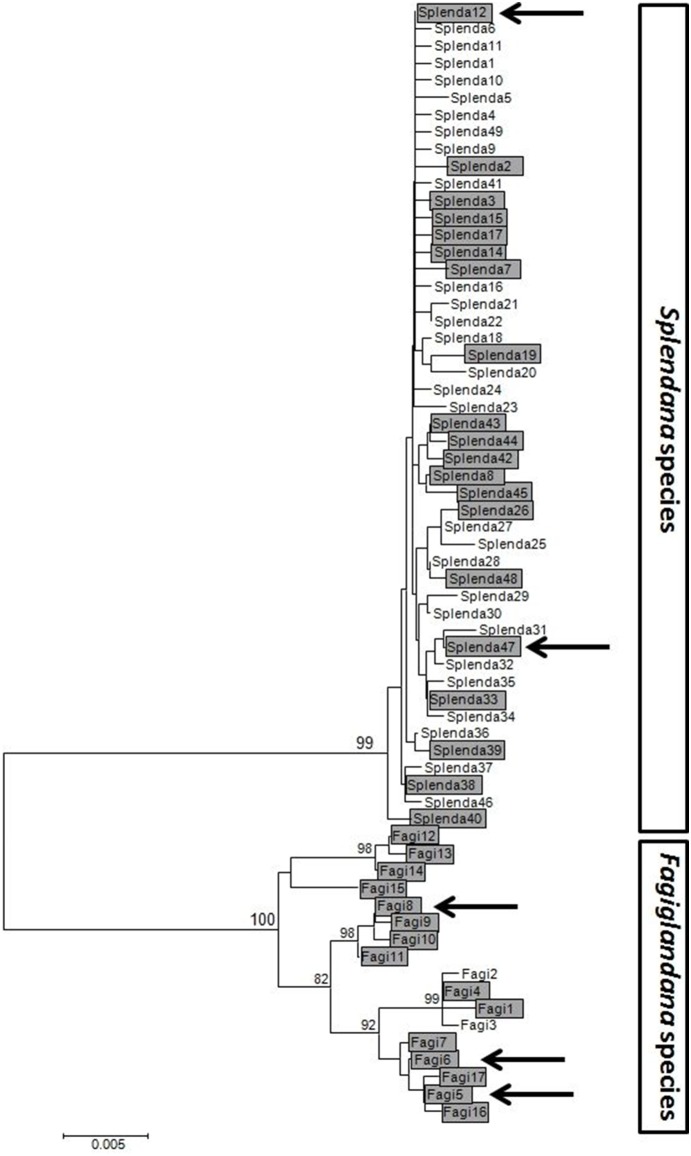
Neighbor-Joining Tree of *Cydia splendana* (Splenda 1–49) and *C. fagiglandana* (Fagi 1–17) haplotypes. Calculations were based on 792 bp of mtDNA COI. Bootstrap support values above 80% are presented above nodes, and the horizontal bar represents 0.005 Tamura-Nei distance. Shaded haplotypes indicate those that contained at least one *Wolbachia* infected individual. Arrows identify the haplotypes that were MLST genotyped.

A 792 bp locus of the mtDNA *COI* gene was analyzed for each of the 243 *Cydia* samples tested for *Wolbachia* infection. Phylogenetic analysis indicated the presence of two distinct clades (Figure 3). The first clade included all samples of *C. fagiglandana*, while all *C. splendana* samples were assigned to the second clade, in complete agreement with the morphological identification. Forty-eight haplotypes were retrieved from the 147 *C. splendana* larvae (Hdiv. = 0.3265), whereas only 17 haplotypes were detected in the 96 *C. fagiglandana* larvae (Hdiv. = 0.1770). Despite the significantly lower haplotype diversity estimated for *C. fagiglandana*, the mean number of single nucleotide polymorphisms (k) was threefold higher compared to the value for *C. splendana* (9.765 versus 3.199 single nucleotide polymorphisms). As a consequence, the mean nucleotide diversity (π) was also threefold higher (0.0123 and 0.0040 for *C. fagiglandana* and *C. splendana*, respectively) (Table 3). Neutral evolution was tested for each population separately and the results are presented in Table 3. In general, estimates of Tajima’s *D* and Fu & Li’s *F* statistics were not statistically significant. While Tajima’s *D* and Fu & Li’s *F* values were statistically supported when calculated for *C. splendana* samples (*D* = −2.48522, P<0.01 and *F* = −4.51468, P<0.02), indicating an excess of rare variants, they were not for *C. fagiglandana* samples (*D* = 0.00147, P>0.1 and *F* = −0.21269 P>0.1) (Table 3). Finally, the mismatch distribution plots of the two species were considerably different, with that for *C. splendana* exhibiting a unimodal curve (Figure S1) whereas the one for *C. fagiglandana* appears ragged (Figure S1).

**Table 3:** Nucleotide polymorphisms of mtDNA *COI* gene and *Wolbachia* infection status of Greek *C. splendana* and *C. fagiglandana* populations.

		*Cydia splendana*							*Cydia fagiglandana*						
			Molecular Diversity				Neutrality tests			Molecular Diversity				Neutrality tests	
		Wolbachia inf	Ht	Hd	π	k	Tajima’sD	Fu &Li’ s F	Wolbachiainf	Ht	Hd	π	k	Tajima’sD	Fu &Li’ s F
**1**	**AO-HAL**	4/4 (100%)	4	1,0000	0,0032	2,5000	−0,79684 NS	−0,75299 NS	12/16 (75%)	7	0,4375	0,0125	9,9048	−0,37846 NS	−0,50512 NS
**2**	**KA-LAR**	2/3 (67%)	2	0,6667	0,0038	3,0000	Cannot be calculated		9/10 (90%)	5	0,5000	0,0119	9,4000	−0,49951 NS	−0,53560 NS
**3**	**ME-DRA**	2/7 (28,6%)	5	0,7143	0,0025	2,0000	0,27345 NS	0,27834 NS	2/7 (22,2%)	5	0,5556	0,0146	11,6000	0,73029 NS	0,78365 NS
**4**	**EA-CRE**	n.d.							4/5 (80%)	3	0,6000	0,0135	10,6667	Cannot be calculated	
**5**	**HO-THE**	1/1 (100%)	1	1,0000	0,0000	0,0000	Cannot be calculated		13/15 (86,67)	5	0,3571	0,0134	10,6000	−0,29593 NS	−0,31777 NS
**6**	**ME-LAR**	3/20 (15%)	11	0,5500	0,0045	3,6000	−1,86291*	−2,50525*	16/19 (84,2)	9	0,4737	0,0119	9,4444	0,57495 NS	1,21707 NS
**7**	**KA-TRI**	5/40 (12,5%)	13	0,3250	0,0028	2,2564	−1,90007*	−2,34303 NS	n.d.						
**8**	**AG-LES**	4/16 (25%)	6	0,3750	0,0042	3,3300	−1,43477 NS	−1,57979 NS	n.d.						
**9**	**SPE-FTH**	3/7 (42,9%)	5	0,7143	0,0023	1,8000	−0,41017 NS	−0,41751 NS	4/4 (100%)	1	0,2500	0,0000	0,0000	Cannot be calculated	
**10**	**HA-EVI**	2/7 (28,6%)	3	0,4286	0,0017	1,3300	Cannot be calculated		n.d.						
**11**	**WE-CRE**	1/2 (50%)	2	1,0000	0,0025	2,0000	Cannot be calculated		3/6 (50%)	2	0,3333	0,0202	16,0000	Cannot be calculated	
**12**	**AN-KAR**	0/5 (0%)	4	0,8000	0,0019	1,5000	−0,75445 NS	−0,67466 NS	3/4 (75%)	2	0,5000	0,0139	11,0000	Cannot be calculated	
**13**	**KA-LAK**	16/28 (57,1%)	8	0,2857	0,0032	2,5357	−1,32001 NS	−1,62334 NS	n.d.						
**14**	**AR-HAL**	0/4 (0%)	3	0,7500	0,0017	1,3300	Cannot be calculated		2/2 (100%)	1	0,5000	0,0000	0,0000	Cannot be calculated	
**15**	**PA-PEL**	3/3 (100%)	3	1,0000	0,0025	2,0000	Cannot be calculated		6/6 (100%)	4	0,6667	0,0116	9,1667	−0,67840 NS	−0,69994 NS
**Total**		46/147 (31%)	48	0,3265	0,0040	3,1990	−2,48522***	−4,51468**	74/96 (77%)	17	0,1770	0,0123	9,7647	0,00147 NS	−0,21269 NS

Haplotypes (Ht) and Haplotype diversity (Hd) were retrieved from the analysis of a 792 bp long locus of mtDNA *COI* gene. Nucleotide diversity (π) and average number of nucleotide differences (k) were based on the number of segregating sites. Neutrality tests were performed only in populations that contained more than 3 individuals, with the following levels of significance: NS: not significant,

*P<0.05,

**P<0.02,

***P<0.01 (n.d.: not detected).

The influence of *Wolbachia* on mitochondrial DNA diversity was also investigated. As shown in Figure 3, *Wolbachia* was detected in 24 out of 51 and in 15 out of 17 haplotypes of *C. splendana* and *C. fagiglandana*, respectively. The infection was fixed in 15 *C. splendana* and 9 *C. fagiglandana* haplotypes, while 6 haplotypes of *C. splendana* and 6 of *C. fagiglandana* included both infected and uninfected specimens. As shown in Table 3, a strong positive correlation was revealed between haplotype diversity (Hd) and *Wolbachia* infection for *C. splendana* (y = 0.3947x+50.97, R^2^ = 0.2935) while for *C. fagiglandana*, this correlation is negative (y = −0.0182x+48.461, R^2^ = 0.0013). The results for the correlation between nucleotide diversity (π) and *Wolbachia* infection were similar. In *C. splendana*, the correlation is still positive but weaker (y = 0.0005x+0.2638, R^2^ = 0.0311), whereas in *C. fagiglandana*, it is strongly negative (y = −0.007x+1.8884, R^2^ = 0.3948).

## Discussion

The detection of *Wolbachia* in *Cydia splendana* and *C. fagiglandana* species infesting chestnuts in Greece, in concert with the pronounced differences in the levels of mtDNA genetic diversity, suggest that the presence of the symbiont might have shaped the population structure of these two species in Greece. Our findings clearly suggest that the species exhibit different *Wolbachia*-infection patterns associated with contrasting mtDNA diversity levels. MLST genotyping also allows a robust separation of *Wolbachia* strains infecting these two species, and indicates horizontal *Wolbachia* transfer between these sister species.

### Wolbachia infection status and mtDNA diversity

Our research adds both chestnut feeding *Cydia* species to the long list of insect species that are infected with *Wolbachia* [52]. *Cydia splendana* as well as *C. fagiglandana* were carrying *Wolbachia* infections; yet the infection status varied significantly between the two species. While *Wolbachia* was found in more than ¾ of *C. fagiglandana* individuals analyzed (77%), the rate was considerably lower for *C. splendana* (only 31%). The high level of *Wolbachia* infection of *C. fagiglandana* is coupled with a reduced haplotype diversity index (H_d_ = 0.1770), compared to that of *C. splendana* (H_d_ = 0.3265). *C. fagiglandana*, in which *Wolbachia* infection is more prevalent, shows a lower mtDNA diversity than the less frequently infected *C. splendana*. Lower mtDNA diversity is attributed to many different factors that range from recent population expansions after bottleneck [46] to selective sweeps [18] and selection against deleterious mutations [53]. The fact that, for both species, no mtDNA haplotype was found to be consistently associated with a specific *Wolbachia* strain for both species [54], along with the fact that several haplotypes contain infected and uninfected individuals, argue against a *Wolbachia*-driven selective sweep.

The strong negative correlation between *Wolbachia*-infection and mtDNA diversity supports the notion that *Wolbachia* has influenced intraspecific divergence, thus reducing the genetic diversity of Greek *C. fagiglandana* populations. This is congruent with previous studies demonstrating a similar effect of *Wolbachia* [18], [55]–[59]. It is interesting to note, however, that the *C. fagiglandana*-clade shows stronger intraspecific divergence, despite harbouring fewer haplotypes. According to Ritter et al. [23], deep intraspecific divergences in DNA barcode studies can be due to both *Wolbachia* infection and phylogeographic structure. However, as for the *C. fagiglandana* individuals, no significant effect of phylogeography could be inferred (both neutrality tests exhibited values with P>0.1 while the mismatch distribution plot was “ragged”, indicating a steady-state population), it can thus be assumed that the dominance of *Wolbachia* rather than other phylogeographic events shaped the intraspecific divergence. For the *C. splendana* individuals in contrast, molecular mtDNA indices (statistically significant negative neutrality tests coupled with a unimodal mismatch distribution plot) argue for population expansion after a recent bottleneck [60]–[61].

### Horizontal transfer

In addition to differentiation at infection level, MLST revealed a further difference in the identity of *Wolbachia* strains infecting the sister species. A strain that belongs to Supergroup B and is identical to the one found in *Colotis amata* (Pieridae, Lepidoptera) from India, was identified in samples of *C. fagiglandana* regardless of geographic origin and genetic assignment. This indicates that *Wolbachia* infection of *C. fagiglandana* was not determined by any other agent, such as environment or intraspecific divergence, something that was already reported before [62]–[63]. On the other hand, most of the *C. splendana* samples were infected with a Supergroup A strain. However, the population PA-PEL was, based on MLST and *wsp*-based analysis, infected with the same strain present in *C. fagiglandana* samples suggesting a possible horizontal transmission event. Several studies have provided evidence that *Wolbachia* strains can be horizontally transferred not only between sister species, but even between distantly related taxa [64]–[65]. By enhancing vertical transmission through horizontal transfer, *Wolbachia* strains increase their potential to spread rapidly and to overcome evolutionary dead-ends that could threaten their survival. Even though it seems to be a rare phenomenon (given the similarities in life cycles and their sympatric distributions), there are still some indications that argue for the scenario of horizontal transfer. The same *Wolbachia* strain infects individuals of different haplotypes, while different *Wolbachia* strains are detected in individuals of the same haplotype. In conclusion, haplotype assignment does not seem to be correlated with the prevalence of *Wolbachia*, which argues for multiple, independent infections [66].

### Distribution of *Wolbachia* infections

At the population level, *Wolbachia* infection varies greatly in both species. The occurrence of *Wolbachia* in *C. fagiglandana* populations ranges from 22.2% to 100%, while in *C. splendana* populations the range was even wider (0–100%). Such strong differences of the infection status between populations have also been recorded in several other arthropod species [63], [67]. The non-uniform infection level among populations is thought to be associated with geographic origin and attributed to local differences in environmental conditions [68]. In our case, the simultaneous study of two sister species from the same sites allows an evaluation of correlation between geographic origin and *Wolbachia* infection status. This approach revealed indeed a weak yet positive correlation (0.1561) between geographic origin of each population and its infection level. As demonstrated in previous studies, the occurrence and prevalence of *Wolbachia* in a given population can depend on environmental conditions, temperature being the stronger effector [69]–[70]. That both species occur broadly in sympatry, and thus are subject to the same environmental conditions, suggests that the *Wolbachia* strains differ in their interactions with these hosts.

## Conclusions

In summary, our investigation reveals the presence of *Wolbachia* in two of the most harmful pests of chestnut production. Screening of several populations of these pests in Greece showed that the prevalence of *Wolbachia*-infection between these species differed significantly, being inversely proportional correlated with mtDNA diversity. The varying *Wolbachia* infection levels among populations of both species suggest an influence of local environmental conditions.

## Supporting Information

Figure S1 Mismatch Distribution diagrams inferred from the haplotypes of Greek *C. splendana* (A) and *C. fagiglandana* (B). The expected frequency is represented by a continuous line, while the observed frequency is shown by a dotted line.
(TIF)

Table S1 Coordinates of the sampled locations.
(DOCX)

Table S2 PCR primers used for *Wolbachia* genotyping.
(DOCX)
