# Supplementary figures and images for: Wolbachia Infections and Mitochondrial Diversity of Two Chestnut Feeding Cydia Species
Source: PLoS One. 2014 Nov 18;9(11):e112795. doi: 10.1371/journal.pone.0112795 (PMC4236127; doi:10.1371/journal.pone.0112795)

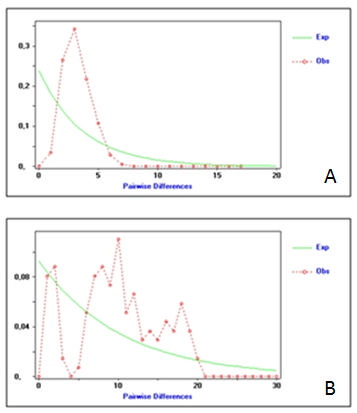

Supplement: Figure S1 — Mismatch Distribution diagrams inferred from the haplotypes of Greek C. splendana (A) and C. fagiglandana (B). The expected frequency is represented by a continuous line, while the observed frequency is shown by a dotted line. (TIF) [file pone.0112795.s001.tif]
