# Supplementary material for: Wolbachia Infections and Mitochondrial Diversity of Two Chestnut Feeding Cydia Species
Source: PLoS One. 2014 Nov 18;9(11):e112795. doi: 10.1371/journal.pone.0112795 (PMC4236127; doi:10.1371/journal.pone.0112795)
Supplement: Table S1 — Coordinates of the sampled locations. (DOCX) [file pone.0112795.s002.docx]

**Table S1. Coordinates of the sampled locations.**

| **Abbreviation** | **Location** | **Longitude** | **Latitude** |
| --- | --- | --- | --- |
| **ME-DRA** | Menoikio (Drama) | 41° 03’ 16’’,35 | 23° 44’ 55’’,06 |
| **HO-THE** | Hortiatis (Thessaloniki) | 40° 38’ 05’’,05 | 23° 07’ 07’’,64 |
| **PA-PEL** | Paiko (Pella) | 40° 57’ 10’’,87 | 22° 20’ 09’’,84 |
| **AR-HAL** | Arnea (Halkidiki) | 40° 29’ 10’’,87 | 23° 35’ 36’’,02 |
| **AO-HAL** | Aghio Oros (Halkidiki) | 40° 19’ 02’’,74 | 24° 04’ 21’’,24 |
| **KA-LAR** | Karitsa (Larisa) | 40° 11’ 10’’,67 | 22° 28’ 56’’,04 |
| **ME-LAR** | Melivoia (Larisa) | 39° 44’ 28’’,85 | 22° 47’ 07’’,86 |
| **AG-LES** | Aghiasos (Lesvos) | 39° 04’ 55’’,86 | 26° 22’ 20’’,37 |
| **SPE-FTH** | Sperchiada (Fthiotida) | 38° 54’ 27’’,05 | 22° 07’ 37’’,95 |
| **AN-KAR** | A. Nikolaos (Karpenisi) | 38° 53’ 17’’,27 | 21° 51’ 31’’,68 |
| **HA-EVI** | Halkida (Evia) | 38° 27’ 58’’,43 | 23° 35’ 27’’,80 |
| **KA-TRI** | Kastanitsa (Tripoli) | 37° 15’ 44’’,49 | 22° 38’ 59’’,78 |
| **KA-LAK** | Kastri (Lakonia) | 37° 09’ 34’’,79 | 22° 18’ 53’’,31 |
| **WE-CRE** | Western Creta (Creta) | 35° 23’ 22’’,79 | 23° 46’ 49’’,39 |
| **EA-CRE** | Eastern Creta (Creta) | 35° 21’ 53’’,94 | 23° 40’ 15’’,40 |
