# Supplementary material for: Wolbachia Infections and Mitochondrial Diversity of Two Chestnut Feeding Cydia Species
Source: PLoS One. 2014 Nov 18;9(11):e112795. doi: 10.1371/journal.pone.0112795 (PMC4236127; doi:10.1371/journal.pone.0112795)
Supplement: Table S2 — PCR primers used for Wolbachia genotyping. (DOCX) [file pone.0112795.s003.docx]

**Table S2. PCR primers used for *Wolbachia* genotyping**

| **Locus code**  **(*w*Mel)** | **Gene** | **Product** | **Name** | **Primer Sequence 5’- 3’** | **Gene length**  **(bp)^a^** | **MLST fragment**  **size (bp)** | **References** |
| --- | --- | --- | --- | --- | --- | --- | --- |
| WD_0146 | *gatB* | glutamyl-tRNA(Gln) amidotransferase, subunit B | gatB_F1  gatB_R1 | GAKTTAAAYCGYGCAGGBGTT  TGGYAAYTCRGGYAAAGATGA | 1,425 | 369 | [53] |
| WD_0301 | *coxA* | cytochrome c oxidase, subunit I | coxA_F1  coxA_R1 | TTGGRGCRATYAACTTTATAG  CTAAAGACTTTKACRCCAGT | 1,551 | 402 | [53] |
| WD_0484 | *hcpA* | conserved hypothetical protein | hcpA_F1  hcpA_R1 | GAAATARCAGTTGCTGCAAA  GAAAGTYRAGCAAGYTCTG | 741 | 444 | [53] |
| WD_0723 | *ftsZ* | cell division protein | ftsZ_F1  ftsZ_R1 | ATYATGGARCATATAAARGATAG  TCRAGYAATGGATTRGATAT | 1,197 | 435 | [53] |
| WD_1238 | *fbpA* | fructose-bisphosphatealdolase | fbpA_F1  fbpA_R1 | GCTGCTCCRCTTGGYWTGAT  CCRCCAGARAAAAYYACTATTC | 900 | 429 | [53] |
| WD_1063 | *wsp* | Outer surface protein | wsp_F1  wsp_R1 | GTCCAATARSTGATGARGAAAC  CYGCACCAAYAGYRCTRTAAA | 714 | 513 | [53] |
| WD_Wp16SA | *16S rRNA* | 16S ribosomal RNA | wspecF  wspecR | YATACCTATTCGAAGGGATAG  AGCTTCGAGTGAAACCAATTC | 1,447 | 438 | [52] |

^a^ With respect to the *w*Mel genome
